# Abdominal Wall Abscess due to Acute Perforated Sigmoid Diverticulitis: A Case Report with MDCT and US Findings

**DOI:** 10.1155/2013/565928

**Published:** 2013-12-09

**Authors:** Rafailidis Vasileios, Gavriilidou Anna, Liouliakis Christos, Tsimitri Asimina, Paschaloudi Sofia, Karadimou Vasiliki

**Affiliations:** Department of Radiology, General Hospital of Katerini, 6 Km Katerini-Arona, 60100 Katerini, Greece

## Abstract

Perforation of the inflamed diverticula is a common diverticulitis complication. It usually leads to the formation of a local abscess. In some rare cases, the inflammatory process may spread towards extra-abdominal sites like the anterior or posterior abdominal wall or the thigh and form an abscess in these sites. We present the case of a 73-year-old man with a history of pain at the lower left quadrant of the abdomen for 20 days and a visible mass in this site. Ultrasonography and computed tomography revealed this mass to be an abscess of the abdominal wall which had been formed by the spread of ruptured sigmoid diverticulitis by continuity of tissue through the lower left abdominal wall. Local drainage of the abscess was performed and the patient was discharged after alleviation of symptoms and an uneventful course. We also discuss causes of abdominal wall abscesses along with the possible pathways by which an intra-abdominal abscess could spread outside the abdominal cavity.

## 1. Introduction

Diverticular disease is estimated to have a prevalence of 20% to 60% and an incidence which increases with age and is rather uncommon in people under 40 years of age. Diverticulitis is the most common complication of diverticular disease and commonly results in perforation of the colon, accounting for 60% of all cases or functional obstruction of the bowel. Perforation of the colon can usually form a diffuse phlegmonous infiltration, a local abscess, or a colovesical fistula [1]. In some rarer cases, extra-abdominal spread of the inflammation process and abscess formation in the abdominal wall may be observed [2].

We present the case of a patient with an abdominal wall abscess which was caused by perforation of inflamed diverticula of the sigmoid colon along with some other cases of extra-abdominal spread of diverticulitis from the literature.

## 2. Case Presentation

A 73-year-old man presented to the emergency department with a history of pain in the left lower quadrant of the abdomen for 20 days. He denied any bowel habit change or being nauseous.

Abdominal examination at presentation revealed a smooth abdomen with tenderness and a clinically visible and palpable mass in the left lower quadrant. Abdominal auscultation revealed normal bowel sounds. Regarding his past medical history, the patient had diabetes, hypertension, chronic obstructive pulmonary disease and had undergone surgery in the prostate and coronary bypass surgery. Laboratory tests showed an increased white blood cell count (14.080/*μ*L) with neutrophilia (77.7%). All other haematological and biochemical indices were within normal range.

Conventional radiography of the chest revealed a small hiatal hernia, whereas abdominal radiography was normal (not presented).

**Figure 1:**
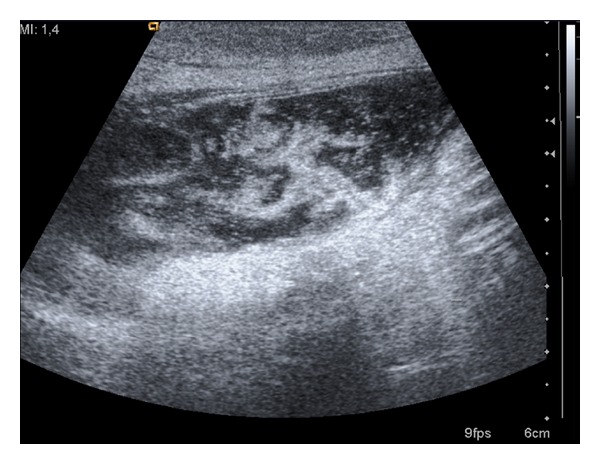
Abdominal wall transabdominal ultrasonography in longitudinal plane with linear probe in trapezoid format showed a mixed echogenicity, oval-shaped space occupying lesion with smooth borders which is situated in the abdominal wall of the left lower quadrant of the abdomen. The echogenic foci situated within the mass possibly represented debris.

The initial thoughts about the differential diagnosis of this abdominal mass included a haematoma, an abscess of the abdominal wall, or some type of hernia. The patient was referred to Radiology Department for an ultrasound examination of the abdominal cavity and the mass. The internal organs were all normal. The ultrasonography of the left lower quadrant of the abdominal wall demonstrated a relatively well-demarcated, oval-shaped mass with mixed echogenicity (relatively more hyperechogenic) whose dimensions were 6.5 × 2.12 cm (Figure 1). The patient was admitted to the Surgical Unit.

**Figure 2:**
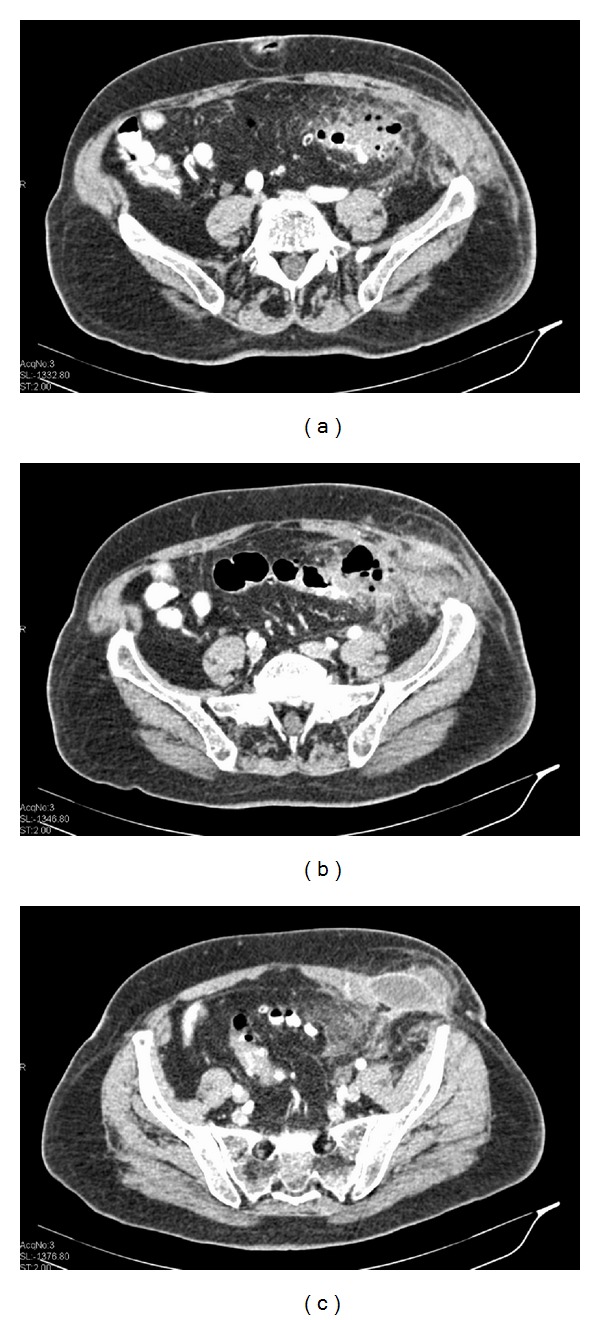
Axial images of contrast-enhanced abdominal MDCT showed wall thickening with narrowing of the lumen and multiple diverticula of the sigmoid colon. There is also pericolic fat stranding, an umbilical hernia (a), thickening of the abdominal wall ((a), (b)), and extraluminal air collection (b). In image (c), we see a bilocular abscess in the lower left abdominal wall and the communication between the abscess and the intra-abdominal inflammation.

**Figure 3:**
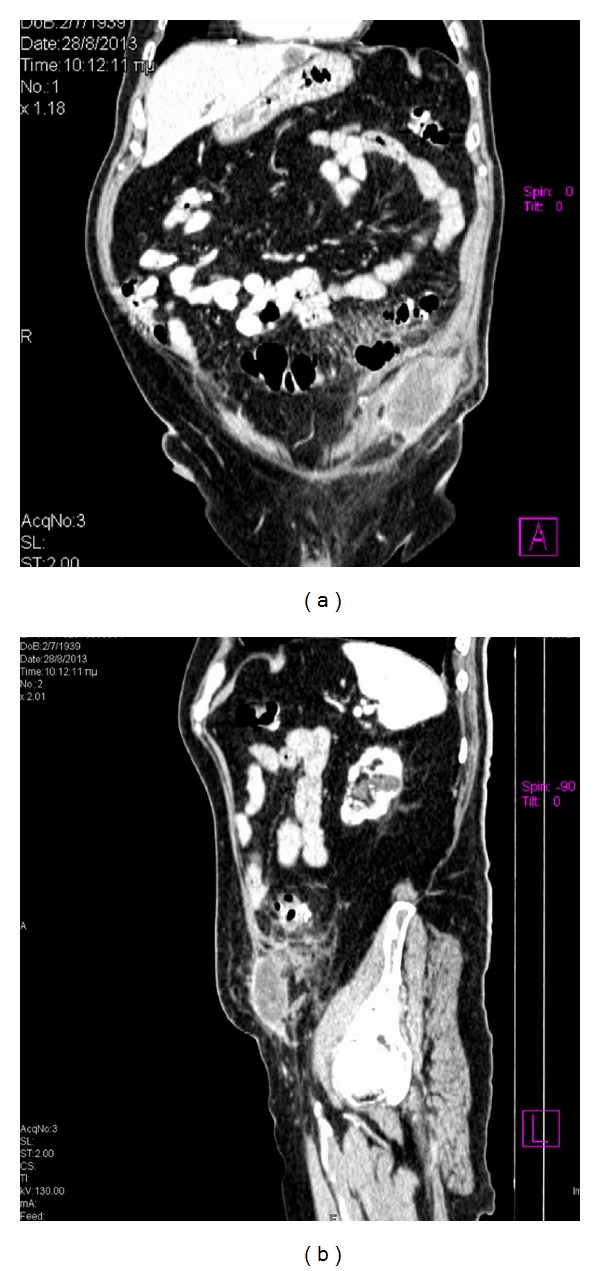
Coronal (a) and sagittal (b) MPR images make more evident the presence of the abscess cavity and its relationship to the abdominal wall, the fat stranding, and the presence of extraluminal free air collection. In image (b), we notice the communication between the abscess and the intraperitoneal inflammatory process.

**Figure 4:**
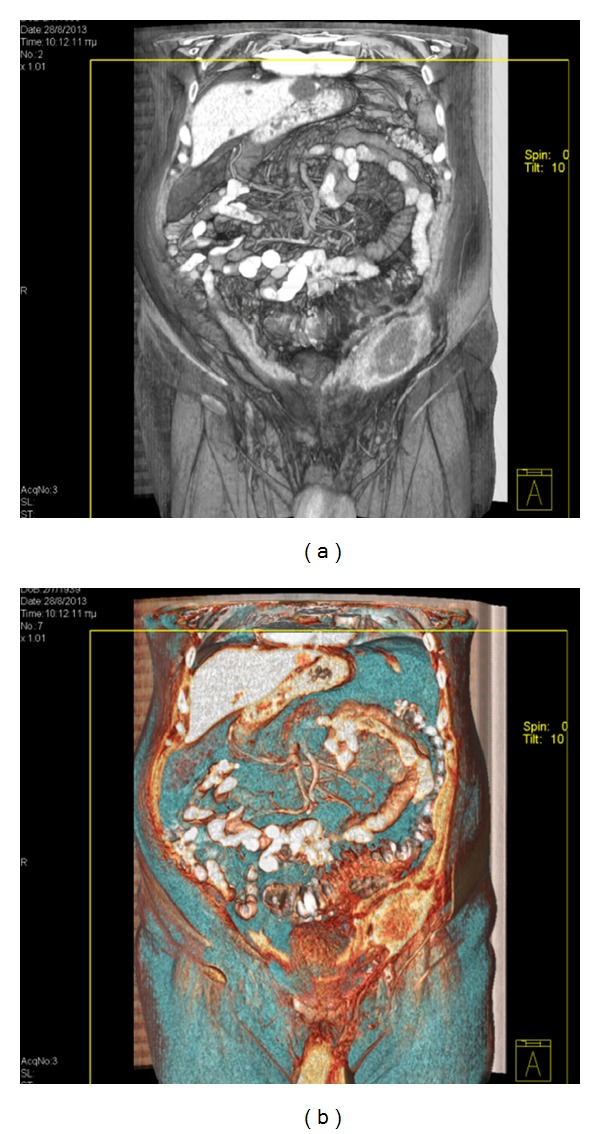
3D volume rendering technique imaging demonstrates the unilateral abdominal wall thickening which is caused by the abscess in relation to other adjacent anatomical structures (a). Image (b) was reconstructed in order to depict the extraluminal air collection in region of the sigmoid colon, in continuity with the abdominal wall abscess.

Contrast-enhanced abdominal multidetector computed tomography (MDCT) was performed the next day of admission and revealed a bilocular abscess inside the lower left lateral abdominal wall. The MDCT also demonstrated inflammation and perforation of multiple sigmoid diverticula with wall thickening of the sigmoid, localised small extraluminal air collection, and fat stranding in the area of the sigmoid. This inflammatory process was located in proximity to the abdominal wall abscess and communicated with it by continuity of tissue. The spread of the inflammation from the abdominal cavity to the abdominal wall was done through the fascia connecting the rectus abdominis with the lateral abdominal muscles. There was also a hiatal hernia containing the stomach and an umbilical hernia (Figures 2, 3, and 4).

**Figure 5:**
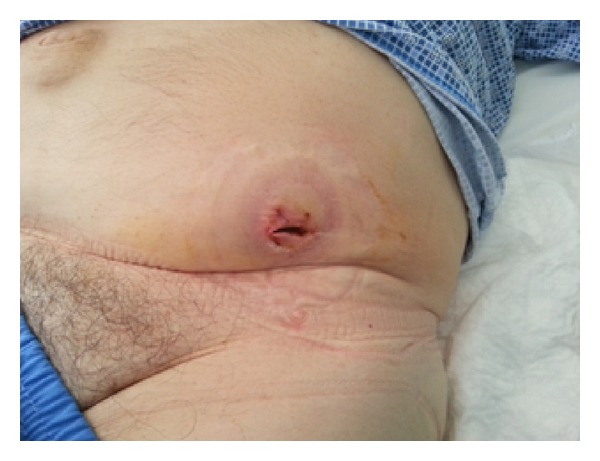
Photograph of the abdominal wall abscess after surgical drainage.

**Figure 6:**
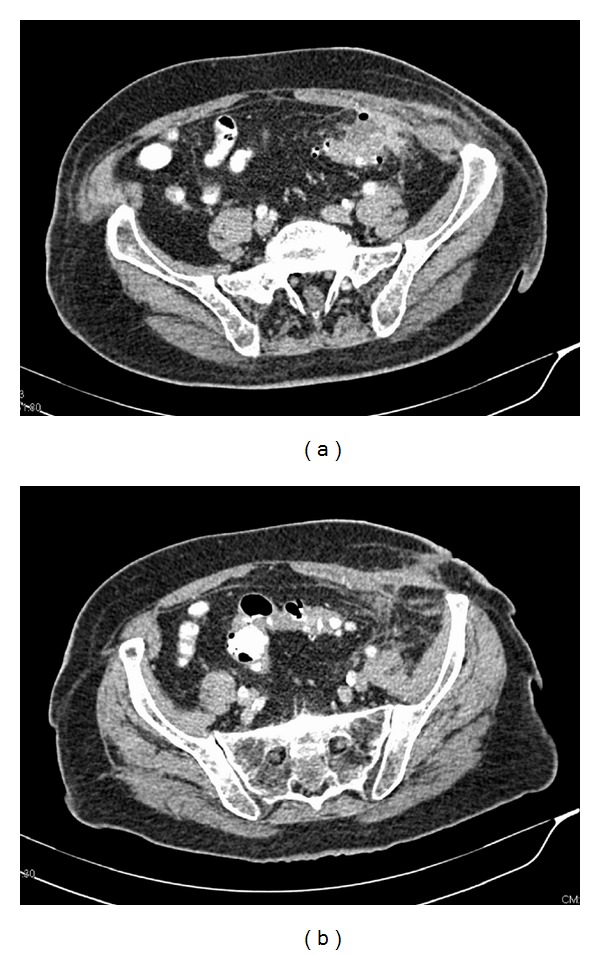
Contrast enhanced abdomen MDCT after surgical drainage of the abscess shows regression of the pericolic inflammation (a), decrease of the quantity of extraluminal air (a), and almost complete regression of the abdominal wall abscess (b). We can also notice the site of drainage in the subcutaneous fat tissue.

Right after the abdominal MDCT, pus was drained from the abscess (Figure 5). During the next days, the patient reported alleviation of symptoms and the second MDCT obtained after the drainage showed regression of the inflammation, decrease of the quantity of extraluminal air, and almost complete regression of the abscess (Figure 6). The patient was discharged after 13 days as his course was uneventful. He was advised for colonoscopy and further followup in the surgical outpatient clinic.

## 3. Discussion

There are several causes of abdominal wall abscesses (AWA). AWAs occur frequently postoperatively, in laparotomy incisions. Other causes of AWA found in the literature are rare and include postoperative gallbladder disease, infections like amoebiasis or non-typhi salmonellosis, malignancies of the transverse colon, xanthogranulomatous pyelonephritis, and diverticulitis of the colon complicated by perforation. In general, inflammatory diseases located inside the abdominal cavity do constitute a cause of AWA. Such intra-abdominal diseases include acute appendicitis, gynecological disorders, Crohn's disease, diverticula of the colon, cholecystitis, and perforation of the intestine. Finally, other AWA may also be caused by iatrogenic causes like placement of catheters, foreign bodies, or rudiments of the urachus [2]. To the best of our knowledge, there is not a recent systematic record of AWA reporting the exact percentage of every cause resulting to AWA.

Perforated diverticulitis may be the cause of extra-abdominal manifestations like abdominal wall abscesses or abscesses in the thigh, hip, and buttock, especially in older patients. Once an abdominal wall abscess is suspected in the differential diagnosis, a CT examination of the abdomen may demonstrate the exact extent of the pathology and possible intra-abdominal diseases accounting for it.

Rothenbuehler et al. reported a case series of 5 patients with inflammatory processes of the abdominal wall and thigh, all caused by diverticulitis. This number of patients was observed during 11 years and after 263 patients had been operated for diverticulitis. These numbers show how rare the extraperitoneal spread of diverticulitis is. Four out of the five patients reported by Rothenbuehler et al. had diverticulitis of the sigmoid like our patient and one had the disease localized in the ascending colon. All patients had abdominal pain for 2 to 8 weeks before hospital admission and increased white blood cell count. Local drainage was performed in all patients and was followed by resection of the affected part of the colon [3].

Stahlgren and Thabit reported in 1961 that the presence of superficial gas in the abdominal wall or thighs can be an indication of unsuspected intra-abdominal or retroperitoneal abscess. This association was first established by Rodlaha in 1926 on a patient with subcutaneous emphysema and sub diaphragmatic abscess caused by perforation of a gastric ulcer. Many reports followed his observation during the following years. In their paper, Stahlgren et al. presented six patients with subcutaneous emphysema and intra-abdominal or retroperitoneal abscesses.

Stahlgren and Thabit also tried to identify the possible pathways by which a paracolic abscess could spread outside the abdomen. They reported seven anatomic routes: (1) along nerves and vessels penetrating the abdominal wall, (2) along the inguinal ring, (3) along the iliopsoas muscle and the femoral vessels to the anterior thigh, (4) through the obturator foramen into the ischiorectal fossa, (5) to the gluteal region and hip through the fossa piriformis, (6) into the perineum along the rectum and finally (7) along Denovillier's fascia to the external genitalia. In our case, we speculate that the inflammatory process spreads to the anterior abdominal wall through the fascia connecting the rectus abdominis muscle and the lateral abdominal muscles, possibly along nerves or small blood vessels [4].

As Stahlgren and Thabit pointed out, one possible pathway of the inflammatory process of diverticulitis is through the psoas muscle to the thigh. Rao et al. reported a case of such a patient with hip pain and subcutaneous emphysema of the left lower limb. This patient's abdominal CT scan revealed an abscess within the psoas muscle which was the result of retroperitoneal perforation of sigmoid diverticulitis which had spread to the left lower limb [5]. Rotstein et al. also published 39 patients with thigh abscess due to diverticulitis and colorectal cancer [6].

Apart from the aforementioned pathways, intra-abdominal inflammations may spread outside the abdominal cavity through “locus of minus resistencia” of the abdominal wall. The lumbar triangle of Petit (or inferior lumbar triangle) is such a site of the posterior abdominal wall. Thus, Coulier et al. reported two cases of gastrointestinal perforations extending through the triangle of Petit and forming extraabdominal lumbar abscesses. The first case was caused by perforated appendicitis and the second was caused by left colonic diverticulitis, as in our patient. Abdominal CT posed the diagnosis in both cases. However, as the authors comment, this kind of intra-abdominal inflammation spread is extremely rare. In general, the retroperitoneal space communicates with the leg along the inguinal ligament, the femoral canal, the sciatic foramen, or the obturator foramen and, through these pathways, retroperitoneal inflammations may spread to the leg [7].

The superior lumbar triangle (or Grynfeltt triangle) also constitutes a weak site of the posterior abdominal wall and thus retroperitoneal inflammations may extend through it to the posterior abdominal wall. Ishigami et al. reported a case of a patient with retroperitoneal appendicitis which extended through the Grynfeltt triangle to the posterior abdominal wall [8].

The formation of an abscess is the second most frequent complication of a perforated cancer of the colon and occurs in 0.3 to 0.4% of the cases. Tsai et al. mention that in half of their patients with perforated colonic cancer, ruptured diverticulitis was the initial diagnosis. This is why attention should be paid in patients presenting with intra- or extraabdominal abscesses. Ultrasonography and computed tomography are of great assistance towards the right diagnosis as they can accurately detect the cause of the abscess preoperatively [9].

Ingestion of a foreign body like a chicken bone is one possible cause of diverticular perforation. It occurs in a small percentage of cases and is asymptomatic until the perforation happens. The site of the perforation may be in the oesophagus, stomach, and small or large intestine and peritonitis is its consequence. Elderly patients, alcoholics, patients with psychiatric diseases, fast eaters, prisoners, people attempting suicide, and other groups of patients are more likely to ingest foreign bodies. Imaging will more likely detect the foreign body when it is metallic. Kornprat et al. conclude by suggesting foreign body ingestion and perforation in the differential diagnosis of diverticular abscess, especially in elderly patients [10].

Another report of intestinal perforation due to foreign body ingestion was done by Erichsen and Sommer. Their patient had ingested a toothpick, albeit not being aware of it. This toothpick caused inflammation in small intestinal loops and formed an abscess in the nearby abdominal wall [11].

When dealing with patients with suspected gastrointestinal perforation, ultrasonography may be helpful. Namely, there are direct imaging findings like an increased echogenicity in the prehepatic space along with comet-tail sign. Indirect signs include fluid collections, thickening of the bowel wall, and ileus. Although the diagnostic accuracy of ultrasonography varies among different studies, some authors conclude that it can be even more sensitive than radiography in detecting gastrointestinal perforation [12].

In a recently published study, the role of MDCT in diagnosing gastrointestinal perforation was evaluated. The authors reviewed CT examinations of patients with surgically proven gastrointestinal perforation and searched for signs of perforation. These signs included the presence of free air, the leakage of oral contrast medium, thickened intestinal wall, wall discontinuity, the formation of abscess, the presence of free fluid collections, and the presence of phlegmon. With the help of these findings, an assumption for the site of perforation was done and was correlated with the surgical findings. The site of perforation was correctly found in a percentage of patients which depended on the part of the gastrointestinal system which was perforated. The overall percentage of correct diagnosis was 82.9%. Free fluid collections and free air were the two most frequent CT findings of gastrointestinal perforation [13].

## 4. Conclusion

Perforation of inflamed sigmoid diverticula is a common complication of diverticular disease which may lead to the formation of a localised abscess. In some rare cases like the one presented, this intra-abdominal inflammatory process may spread outside the abdominal cavity and cause the formation of extra-abdominal abscesses.
