# Erratum to “Abdominal Wall Abscess due to Acute Perforated Sigmoid Diverticulitis: A Case Report with MDCT and US Findings”

**DOI:** 10.1155/2014/493515

**Published:** 2014-03-12

**Authors:** Vasileios Rafailidis, Anna Gavriilidou, Christos Liouliakis, Asimina Tsimitri, Sofia Paschaloudi, Vasiliki Karadimou

**Affiliations:** Department of Radiology, General Hospital of Katerini, 6 Km Katerini-Arona, 60100 Katerini, Greece

In the paper, the authors' names were mistakenly cited in a wrong format (family name first, first name second). We herein cite the authors' names in the correct format (first name first, family name second).

